# It Sounds like It Feels: Preliminary Exploration of an Aeroacoustic Diagnostic Protocol for Singers

**DOI:** 10.3390/jcm12155130

**Published:** 2023-08-04

**Authors:** Calvin Peter Baker, Suzanne C. Purdy, Te Oti Rakena, Stefano Bonnini

**Affiliations:** 1 Speech Science, School of Psychology, University of Auckland, Auckland 1023, New Zealand; calvin.baker@auckland.ac.nz; 2 School of Music, University of Auckland, Auckland 1010, New Zealand; t.rakena@auckland.ac.nz; 3 Department of Economics & Management, University of Ferrara, 44121 Ferrara, Italy; bnnsfn@unife.it

**Keywords:** functional diagnostics, preventative healthcare, self-perception, vocal fatigue, singing voice analysis

## Abstract

To date, no established protocol exists for measuring functional voice changes in singers with subclinical singing-voice complaints. Hence, these may go undiagnosed until they progress into greater severity. This exploratory study sought to (1) determine which scale items in the self-perceptual Evaluation of Ability to Sing Easily (EASE) are associated with instrumental voice measures, and (2) construct as proof-of-concept an instrumental index related to singers’ perceptions of their vocal function and health status. Eighteen classical singers were acoustically recorded in a controlled environment singing an /a/ vowel using soft phonation. Aerodynamic data were collected during a softly sung /papapapapapapa/ task with the KayPENTAX Phonatory Aerodynamic System. Using multi and univariate linear regression techniques, CPPS, vibrato jitter, vibrato shimmer, and an efficiency ratio (SPL/P_Sub_) were included in a significant model (*p* < 0.001) explaining 62.4% of variance in participants’ composite scores of three scale items related to vocal fatigue. The instrumental index showed a significant association (*p* = 0.001) with the EASE vocal fatigue subscale overall. Findings illustrate that an aeroacoustic instrumental index may be useful for monitoring functional changes in the singing voice as part of a multidimensional diagnostic approach to preventative and rehabilitative voice healthcare for professional singing-voice users.

## 1. Introduction

The human voice is a versatile instrument that allows for the transmission of complex data including societal traditions, histories, codes, and emotions. Only small changes in voice production are needed to produce great shifts in intent and meaning. Professional singers rely on subtle and nuanced changes in voice function that require mobile, robust, and healthy vocal folds. Deterioration in voice production may significantly impact quality of life when a singer’s voice is affected by organic (structural or neurological) or functional disorders [1,2,3]. Reputation in the artistic community and ability to earn a livelihood can also be negatively affected [4].

For voice researchers, clinicians, and pedagogues, singing-voice analysis presents unique challenges. Many of the widely used voice assessment techniques (e.g., local pitch and amplitude perturbation measures) rely on methods that may not be robust to singing-voice variables such as wide ranges in *f*_o_, intensity, or vibrato characteristics (e.g., [5,6,7,8,9]). Additionally, traditional clinical voice analysis tasks (e.g., sustained vowels and reading passages at a comfortable pitch and intensity) do not incorporate the singing voice or consider singing-specific phenomena such as registration events or vibrato characteristics. While some may reason that speech samples are sufficient for all voice analyses, the analogous idea of analyzing task-specific movements of elite athletes without having them perform tasks relevant to their professional context is incongruous. If a singer presents with a singing-voice complaint, their singing voice should be analyzed.

Non-traumatic (i.e., not caused by a specific injury or event) clinical voice disorders (e.g., muscle tension dysphonia or nodules) are preceded by functional changes that increase risk of vocal injury [10,11]. Even without clear visual findings, maladaptive changes in vocal function result in inefficiencies and discomfort that are readily perceived by the trained voice user [12,13]. Therefore, determining biomarkers of early functional voice disorder is critical for preventative and habilitative healthcare for professional singing-voice users. Instruments that can measure functional changes in the singing voice related to singing-voice complaints may improve methods for monitoring vocal health through periods of hormonal or physiological change or periods of increased vocal demand (e.g., intense performance runs or leading up to performance exams). Patel et al. [14] recommend protocols for speech analysis but do not comment on the singing voice or the unique challenges related to singing voice analysis. To date no standardized protocol exists for quantitative singing-voice analysis in a clinical context, suggesting that these subclinical voice complaints must progress into greater severity before treatment is offered, i.e., when potential livelihood is impacted.

The Evaluation of Ability to Sing Easily (EASE) [15] was developed in acknowledgement of the unique voice complaints experienced by singers. The EASE is a self-rating scale consisting of three subscales that should be scored and interpreted separately: Vocal Fatigue (*VF*), Pathological Risk Indicators (*PRI*), and Voice Concerns (*VC*). The final instrument is a 22-item questionnaire using the four-point Likert-type responses *Not at all* (1); *Mildly* (2); *Moderately* (3); and *Extremely* (4). Appendix A provides a full list of the 22 items. Phyland (2014, unpublished data) reported good internal consistency for each subscale (Cronbach’s 𝛼 all > 0.8) and statistically significant correlations (*p* < 0.001) between each of the subscales. The EASE has shown promise in distinguishing between healthy and disordered singers and appears to be sensitive to subtle functional changes perceived by professional and semi-professional voice users [16,17,18,19,20]. The EASE is unique in that it was constructed to measure self-perceived vocal status without the assumption of voice disorder or injury [15,21], making it a particularly relevant tool for use with singers with subclinical voice complaints. While it has recently been recommended as part of a multidisciplinary approach when working with singers in the voice clinic [22], few studies have explored associations between EASE subscale items and instrumental voice measures.

### 1.1. Instrumental Analysis of the Singing Voice

Although there are many acoustic measures to choose from for speech-level analysis, fewer have proven efficacy for use with sung samples. Inverse filtering is a useful method for extracting voice-source information for both spoken and sung samples. The non-invasive nature of inverse filtering an acoustic signal allows singers to perform sung vocal tasks generally unencumbered. Inverse filters (or antiresonances) are used to counteract the vocal-tract transfer function, leaving only the estimated voice-source spectrum and the flow-glottogram (FLOGG). The normalized amplitude quotient (NAQ) is one parameter that can be calculated from the FLOGG and its first derivative [23,24]. It reflects the degree and quality of glottal closure related to phonation type, from breathy to pressed, and between singing styles [23,25,26,27,28]. The NAQ operates in the amplitude domain and hence is less affected by glottal event delineation [23,29]. As the NAQ infers glottal configuration related to phonation type, there may be an association between EASE subscale scores and NAQ values. One limitation is that the successful extraction of the FLOGG depends on accurate determination of the first two formants (*F*_1,2_). Conveniently, the inverse filter module of *Sopran* [30] contains a real-time display of the FLOGG as the inverse filters are applied, allowing the user to adjust the frequencies to ‘tune’ the inverse filters to achieve a ripple-free closed phase in the FLOGG and a smooth source-spectrum tilt with no large dips surrounding the formants.

Relative average perturbation (RAP, %) and amplitude perturbation quotient 3 (APQ3, %) quantify pitch and amplitude perturbation in the glottal cycle, smoothed across three consecutive periods. They have been used successfully with singing voice samples [31,32] and are not widely affected by changes in *f*_o_, intensity, or vibrato extent (VE) as are local jitter and shimmer (Baker et al., in review). An increase in EASE subscale scores may also be reflected in increased in RAP, APQ3, or both.

Smoothed cepstral peak prominence (CPPS) reflects the dB difference between the cepstral peak (most prominent rahmonic in the cepstrum), and a linear regression line at the same quefrency (ms). CPPS successfully discriminates between dysphonic and normophonic voices and has shown sensitivity to breathiness in normophonic speakers [33,34,35,36,37]. As CPPS is robust to factors such as environmental noise and microphone selection [38], it may be a useful clinical tool for tracking subtle changes in the singing voice. After controlling for the effects of *f*_o_ and intensity [7,39], a decrease in CPPS values may be associated with elevated EASE subscale scores.

The ubiquity of vibrato in the Western classical singing voice (WCSV) makes it a highly relevant candidate for singing-voice analysis in classically trained singers. Systematic contemporary commercial music (CCM) voice pedagogy is relatively young [40,41]. However, the present authors note growing consensus among practitioners that, while stylistic choices may influence vibrato characteristics, a well-balanced ‘neutral’ vocal production (i.e., not shaped by stylistic voice effects) that includes a stable and free vibrato should be a goal for CCM singers, from which artistry can be shaped. Stability in vibrato rate (VR) and VE is dependent on stable oscillatory mechanisms and fine intralaryngeal muscle coordination [42,43,44]. Morelli and colleagues [45] presented the *BioVoice* voice-analysis software that includes two measures for singing voice vibrato perturbation analysis: vibrato jitter and vibrato shimmer (hereafter V_Jitt_ and V_Shim_). Like the well-known jitter and shimmer measures that measure frequency and amplitude perturbation in the acoustic waveform, V_Jitt_ and V_Shim_ quantify perturbation in the *f*_o_ vibrato waveform of a sung sample (for V_Jitt_ and V_Shim_ equations see [46]). Vibrato is a multidimensional phenomenon that includes cyclical muscular contractions producing a quasi-sinusoidal *f*_o_ oscillation. As such, inefficiencies in function or structural changes in the vocal folds are likely to result in decreased stability of VR and VE. Thus, the severity of singer-perceived voice complaints may increase with higher V_Jitt_ and V_Shim_.

Aerodynamic measures provide complementary data to acoustic analyses that have clear physiological attributes. The Phonatory Aerodynamic System (PAS) [47] provides information on inferred subglottal pressure (P_Sub_; cm H_2_O, measured from intra-oral pressure during a /p/ occlusion), airflow during voicing (l/s), and sound-pressure-level (SPL [dB]). These data can be used to calculate various efficiency ratios. As described by Toles et al. [48], the SPL-to-P_Sub_ ratio decreases with incomplete adduction, perhaps due to functional or structural changes [49,50]. Thus, lower efficiency ratios may be associated with higher EASE subscale values. In this study, the ratio used will be referred to as Efficiency Ratio (ER), and is defined in Equation (1):

(1) $$\mathrm{ER}=\frac{\mathrm{SPL}}{P_{\mathrm{Sub}}}=\frac{\mathrm{dB}}{\mathrm{cm}\;H_{2}O}$$

While the measures explored here are not exhaustive, they offer complementary data on vocal function, can be successfully computed from sung samples, and can be easily implemented using existing tools in clinical and research contexts.

### 1.2. The Present Study

To date little research has been carried out exploring the links between instrumental voice measures and singers’ perceptions of their own vocal function and health. These are needed to help determine biomarkers that indicate at-risk vocal function in professional singing-voice users with voice complaints and are critical for developing evidence-based preventative and rehabilitative healthcare approaches for singers.

The present exploratory study sought to (1) examine associations between selected acoustic and aerodynamic instrumental measures and the individual scale items of the EASE, and (2) to develop as proof-of-concept a multi-instrumental quantitative index of biomarkers that is sensitive to singers’ self-perceived vocal function and health status. Instrumental measures were selected a priori based on their suitability for singing voice analysis and proven efficacy for tracking functional changes in voice behavior. We predicted that higher EASE item values would be associated with reduced vocal stability and efficiency as gauged through acoustic and aerodynamic voice measures.

## 2. Materials and Methods

### 2.1. Participants

A cross-sectional cohort of healthy cisgender male and female singers was recruited by a third party from the University of Auckland, School of Music Classical Voice Department. As the trans voice presents unique variations in function and may be structurally altered by hormonal or surgical intervention [51,52,53,54], only cisgender singers were included in this study. However, further specific research on non-binary and trans voice is needed. All singers were classically trained and had experience performing in solo, choral, and ensemble contexts. Data were collected between March and August 2022.

The singers were first asked to complete one online questionnaire which included the Singing-Voice Handicap Index-10 (SVHI-10) as a screening tool [55], and demographic data including self-reported ethnicity, stage of study, and total years of training. Participants were asked to disclose any previously diagnosed vocal injury or hearing loss and were seen by a laryngologist to assess vocal health and function. Female participants’ recording sessions were scheduled to avoid the pre and perimenstrual period [56,57,58].

### 2.2. Acoustic and Aerodynamic Recordings

Each participant was first given five minutes alone in a sound-treated room to warm up their singing voice [59,60] and was asked to perform warmup tasks as if they were preparing for a solo performance. Following the warmup, participants were seated in the room with the researcher for recording. A headset omnidirectional condenser microphone (AKG HC 577L; AKG Acoustics, Vienna, Austria) positioned 7 cm adjacent (45°) to the right of the participant’s mouth was used to capture the acoustic voice signal. The microphone was connected to a MacBook Pro running *PRAAT* v. 6.2.16 [61] via a pre-amplifier (MobilePre [MK II]; M-Audio, Rhode Island, USA). All recordings were captured at a 44.1 kHz sample rate. Participants were asked to sustain an /a/ vowel at any comfortable pitch and intensity, during which the C-weighted SPL was measured using an SPL-meter held adjacent to the microphone position. The SPL (*L*_Ceq_) was announced by the researcher into the microphone to use later for dB SPL signal calibration before acoustic analyses [62].

Aerodynamic measurements were made using the PAS, a handheld device containing a transducer system that records airflow (through a mask), intra-oral pressure (through an intra-oral tube), and an acoustic signal. The microphone is fixed at a standard distance of approximately 15 cm from the mouth (5 cm preset position). Flow (l/s), pressure (cm H_2_O), and SPL (dB) data were captured simultaneously in real time during the consonant-vowel (CV) train /papapapapapapa/.

### 2.3. Sung Tasks

Following warmup and calibration, participants were asked to sing a quiet /a/ vowel using their usual performance technique on C4 (261.63 Hz, low voice types) or C5 (523.25 Hz, high voice types). The starting *f*_o_ for each task was sounded on a digital keyboard before each attempt. Singers were asked to ensure the tone was sung as quietly as possible, whilst maintaining a solo-performance standard of volume, i.e., not a whisper. Soft (but not whispered) phonation requires a fine balance of P_Sub_ and glottal adduction [63,64,65], and therefore may be more useful in demonstrating vocal-fold related issues such as fatigue or oedema, which can be disguised by louder voicing, when the vocal-folds are more tightly adducted. High voice types were also asked to sing the same vowel on C4, which could be used later during inverse filtering as an approximate reference for *F*_1_ and *F*_2_ if necessary. After these tasks, participants completed the full EASE based on their voice production during the warmup and recording session only.

Following the acoustic recordings and completion of the EASE, participants were instructed how to use the PAS device. They were then asked to sing the CV train /papapapapapapa/ on C4 (261.63 Hz, low voice types) or C5 (523.25 Hz, high voice types) in one breath as quietly as possible without whispering, in a similar manner to the acoustic recording. Raw data were visually inspected to ensure that the intra-oral pressure value returned to 0 cm H_2_O during vowel phonation. The first and last utterances were discarded, and the averaged numerical data of the remaining five utterances were saved to a text file.

### 2.4. Acoustic Data Processing

Each participant’s acoustic recording was saved in its entirety as a .wav file. Tasks were then separated and saved as individual files for analysis. The most stable medial five-second portion of the soft phonation sung task was used for acoustic analysis. Selections were made at the nearest zero-crossings and were checked for clipping, distortion, or extraneous noise aurally and through spectrographic review.

The trimmed acoustic signal was imported into *Sopran* [30] and calibrated with respect to SPL using the calibration tone collected at the time of recording [62]. The NAQ was obtained by first re-sampling the signal to 16 kHz, then inverse filtering the most stable one-second portion of the sung tone. As all singers performed an /a/ vowel, a reasonable estimate of the locations of *F*_1_ and *F*_2_ was possible based on a priori knowledge [66,67,68]. The inverse filters were tuned to obtain a waveform ideally with a ripple-free zero-flow phase in the FLOGG and a source-spectrum slope free from peaks or troughs surrounding formants [26,69,70]. If a zero-phase was not apparent (likely due to incomplete glottal closure), the inverse-filtered spectrum and negative peak of the flow derivative were used as guides for filter tuning [69,70]. If necessary, the C4 tone produced by the high voice type singers was used as a starting point for tuning formant frequencies. All data were checked for outliers and the process was repeated if a participant’s NAQ values were well outside previously reported norms, i.e., 0.1–0.3 [23,25,29].

The CPPS was calculated in *PRAAT* v.6.2.16 [61] using the ‘To PowerCepstrum’ and ‘Get CPPS’ functions as described in earlier works [9,71,72]. All settings were kept as standard [61] apart from ‘Peak search pitch range (Hz)’ which was increased to 1000 Hz to ensure the *f*_o_ of all tasks were well accommodated [7]. The RAP and APQ3 were obtained in the ‘voice report’ function of *PRAAT* using standard settings. The freeware *BioVoice* [45] was used to calculate two measures of vibrato regularity for each signal: V_Jitt_ and V_Shim_. Numerical results were saved in an *Excel* file after automatic analysis and then integrated into the combined data set.

### 2.5. Statistical Analyses

Data were statistically analyzed in *RStudio* v. 4.2.1 [73]. Box plots and histograms were used to explore the data and determine the presence of outliers. Multicollinearity was assessed using the variance inflation factor (VIF; Equation (2)), whereby each predictor variable was entered into a separate multiple linear regression model as the dependent variable and tested against the other predictors [74,75]. The VIF numerical threshold for variable inclusion was <5 [75].

(2) $$\mathrm{VIF}=\frac{1}{1-R^{2}}$$

Multivariate regression and Pillai’s trace with backward elimination were used to determine which instrumental measures (i.e., NAQ, RAP, APQ3, CPPS, V_Jitt_, V_Shim_, and ER) were associated with the individual EASE scale items. This approach allowed for the joint estimation of all coefficients and the evaluation of single effects in relation to all others. A composite score was then calculated from these scale items. Kendall’s tau-b was used to determine the strength of association between the reduced-item scale and the *VF*, *PRI*, and *VC* subscales of the original EASE tool, respectively. To reduce the effect of possible Type-II errors arising from a small sample, the significance level was set at 0.10.

Predictor variables with a VIF < 5 were included in regression models [76], as well as gender, age, years of training, *f*_o_, and SPL. Multiple linear regression was carried out using a backward elimination iterative method where predictor variables were systematically removed from the model using the largest *p*-value as criteria for exclusion in each iteration. The process was repeated until only predictor variables with *p*-values less than 0.10 were included [74,75]. Finally, multivariate normality was confirmed through non-significant skewness and kurtosis in the models’ residuals and Mahalanobis’ distances [77,78].

## 3. Results

Nineteen singers volunteered for participation (soprano [7], mezzo-soprano [1], alto [1], tenor [4], baritone [5], and bass [1]). The mean SVHI-10 score (*M* = 10.89; *SD* = 5.28) was higher than norms recorded by Sobol et al. [79], and one participant disclosed a history of diagnosed vocal injury. Their data was excluded from the ensuing analyses. The remaining 18 participants underwent visual inspection of the vocal folds by a laryngologist and were free of functional or organic voice disorder. Participants’ mean age was 26.61 years (*SD* = 8.94, range = 19 to 59 years). Reported ethnicities included European (2), NZ European (9), Asian (3), Māori (3), and Pasifika (1). Mean years of lessons at a tertiary level was 9.94 (*SD* = 7.67, range = 1 to 37 years). Table 1 shows the descriptive statistics for included instrumental variables. In testing for multicollinearity, only RAP had a VIF greater than 5 and so was removed from ensuing analyses.

Multivariate regression with backwards elimination revealed three scale items that were significantly associated (*p* < 0.10) with instrumental measures: Q1 ‘My voice is husky’; Q2 ‘My voice is dry/scratchy’; and Q11 ‘My top notes are breathy’. No age, gender, or training effects were found. The composite values for these three scale items are henceforth referred to as the EASE-3. The EASE-3 had a mean value of 5.17 (range = 3 to 8, *SD* = 1.65) out of a possible 12, where 3 indicates no difficulty at all and 12 indicates an extreme level of difficulty. Construct validity was tested against the original EASE *VF*, *PRI*, and *VC* subscales using Kendall’s tau-b. A strong, statistically significant associations were seen between the *VF* subscales and the EASE-3 (𝜏 = 0.742, *p* < 0.0001). The *PRI* subscale was moderately associated with the EASE-3 (𝜏 = 0.324, *p* = 0.089). No correlation was found between the *VC* subscale and the EASE-3 (𝜏 = 0.046, *p* > 0.10).

Using Pillai’s trace tests with backwards elimination, four significant coefficients’ estimates were revealed (all *V* > 0.55, *p* < 0.05), corresponding to the explanatory variables CPPS, V_Jitt_, V_Shim_, ER. A univariate model including these measures showed a good fit for the EASE-3 data and was statistically significant, adjusted R^2^ = 0.624, *p* < 0.01. Residual skewness and kurtosis for this model were non-significant (*p* > 0.05) and Mahalanobis’ distance was below the critical D^2^ value of 27.69 (12.88, *p* < 0.01). Signal SPL (dB), *f*_o_, age, gender, and years of training showed no contribution in explaining variance, *p* > 0.10. The regression model for the EASE-3 is shown in Table 2. The regression equation is presented in Equation (3). Values derived from the model had a strong correlation with the original *VF* subscale (𝜏 = 0.575, *p* = 0.001), a moderate correlation with the *PRI* subscale 𝜏 = 0.295, *p* = 0.098), and no correlation with the *VC* subscale (𝜏 = 0.070, *p* > 0.10).

(3) $$\hat{y}=14.0114-0.41753*CPPS+0.10822*V_{Jitt}+0.1164*V_{Shim}-0.6764*ER$$

## 4. Discussion

Trained signers are sensitive to subtle changes in voice function that may not be apparent under visual examination. This does not mean, however, that these complaints should be taken lightly or dismissed; these subclinical functional changes may be precursors to developing functional or organic voice disorders such as muscle-tension dysphonia or space-occupying mass (e.g., nodules). To date, no established clinical protocols exist for working with the singing voice, and few studies have considered the suitability of traditional voice analysis techniques for singing voice analysis. This suggests that a singer’s voice complaint must increase in severity (i.e., into dysphonia) before it is quantitatively measurable using clinical diagnostic instruments with speech samples. This is too late for the professional voice user who relies on optimal vocal function for livelihood. Furthermore, delay in diagnosis of subclinical functional disorders may lead to anxiety and loss of confidence and self-efficacy [80,81,82,83].

The EASE was developed to collect data on singers’ self-perception of their vocal function and health at a single time point [15,21]. The EASE and its subscales have shown promise in distinguishing dysphonic from normophonic singers, and in measuring singers’ perceptions of vocal function and health during periods of high vocal demand and in pre/postintervention studies [17,84,85]. We initially hypothesized that an increase in singers’ EASE scores would be associated with increased values in acoustic measures, and decreased ER. Multiple linear regression with backwards elimination determined four instrumental predictors (CPPS, V_Jitt_, V_Shim_, and ER) that were significantly associated with three of the original 22 scale items: (1) My voice is husky, (2) My voice is dry/scratchy, and (3) My top notes are breathy. The significant association (𝜏 = 0.742, *p* < 0.0001) found between the combined EASE-3 score and the *VF* subscale of the original EASE supports that the EASE-3 primarily reflects biomarkers of vocal fatigue in the singing voice [15]. The significant relationship (*p* = 0.001) between the instrumental index and the original *VF* subscale in our data suggests that the development of a protocol and instrumental index for diagnosing and tracking vocal fatigue and effort-related symptoms in the singing voice is feasible. Given this association, we have termed the instrumental model constructed in this study the Aeroacoustic Singing Fatigue Index (ASFI).

### 4.1. Symptoms of Vocal Fatigue and the EASE-3

Hunter et al. [86] define vocal effort as the ‘perceived exertion of a vocalist to a perceived communication scenario’ (p. 516), and vocal fatigue as ‘a quantifiable decline in function’ (p. 516). Vocal effort is a commonly reported complaint for professional voice users in many sectors including performance, telemarketing/health, and education [4,13,87]. Reported symptoms of increased perceived vocal effort and measurable vocal fatigue include increased instability and breathiness, reduced agility and range, laryngeal discomfort, and increased phonation threshold pressures (PTP) [88,89,90]. The etiology of these symptoms is multifaceted and may arise (for example) from changes in vocal-fold viscosity, fatigue of intralaryngeal musculature and connective tissue, dehydration, or a combination of factors including these [91,92]. The nearly ubiquitous manifestation of vocal fatigue in functional, structural, and neurological dysphonia highlights its clinical significance [93]. There are clear connections between functional or organic pathologies and perceptual experiences of increased vocal effort. However, increased vocal effort and discomfort may also be present in the absence of visually identified pathology [94].

Vocal fatigue has an intuitive relationship with vocal demand and vocal demand response. Increased duration and intensity of vocal fold vibration during prolonged speech or singing incurs greater impact stress during vocal fold collision. Increased tissue viscosity in the vibrating portion of the vocal folds and reduced ability to mitigate the resulting increased friction (i.e., heat energy) have been proposed as contributing factors to vocal fatigue [91]. Despite these seemingly clear characteristics, few studies have found significant correlations between perceptions of vocal fatigue and instrumental voice measures; studies that have investigated this seem to present varied conclusions [87,95,96,97]. To the authors’ knowledge, no research in this area has been carried out with a focus on the singing voice.

The items included in the EASE-3 have clear connections with known symptoms of vocal fatigue and functional disorder such as huskiness, dryness, scratchiness, and strain [98], some of which have also been included in the widely used Vocal Tract Discomfort Scale [12,99,100,101]. In the EASE-3 these sensations are reported in Q1 (My voice is husky) and Q2 (My voice is dry/scratchy). Breathiness is also part of the symptomology of vocal effort and fatigue [89] and is easily recognized by both singer and listener. Glottal sufficiency and its relation to breathiness is implied in scale item 11 (My top notes are breathy). For singers, the quality of high notes is particularly enlightening. Singing effectively at high frequencies requires fine coordination of aerodynamic and muscular function for optimal phonation that exposes the condition of the voice in a way that conversational speech may not. The third item in the EASE-3 (Q11 in the full EASE) relates directly to breathiness when singing high notes. Together, the EASE-3 is comprised of questions related to known traits of vocal fatigue and functional disorder and offer singer-specific contexts that are vital when analyzing the signing voice. We are not suggesting that the EASE-3 replace the original EASE *VF* subscale, however, in our data only these three questions offered psychometric data that could be related to quantitative aeroacoustic measures.

### 4.2. Perceptions of Singing Vocal Fatigue and Acoustic Measures

Acoustic voice measures offer instrumental (quantitative) and non-invasive insights into vocal function during phonation. However, few have been related to self-perceptual measures of vocal function and fatigue. The ASFI presented here includes CPPS, V_Jitt_, and V_Shim_, which, respectively, can be related to symptoms of vocal fatigue.

In our data, participants’ CPPS values ranged from 11.58 to 18.02 dB (*M* = 14.4, *SD* = 1.88), which are within previously reported ranges for healthy speakers [39,102]. In previous research, Saeedi et al. [103] found associations between cepstral measures (CPP and CPPS) and elements of two different self-perceptual vocal health tools: the Vocal Tract Discomfort Scale (Persian) and the Non-Standard Hoarseness Self-Assessment. Their findings suggest that CPPS reflects some element of phonation that is directly perceivable by the speaker (or singer). Bhuta et al. [104] reported correlations between other noise-related measures (Noise-to-Harmonics Ratio [NHR], Voice Turbulence Index [VTI], and Soft Phonation Index [SPI]) and the perceptual Grade, Roughness, Breathiness, Aesthenia, Strain (GRBAS) scale recorded from 37 dysphonic speakers. These associations between CPPS, breathiness, and perceptual voice analysis support the current findings.

The presence of breathiness in the voice is a readily-percievable voice characteristic that classically trained singers typically work to eliminate [64,105]. CPPS offers insights into the presence of turbulent breath noise in the voice signal, and is strongly related to voice source behaviour. It may be that fatigue of vocal-fold adductor muscles or swelling of the vocal folds themselves contribute to incomplete glottal adduction or a non-simultaneous closing phase that increases noise components in the signal (i.e., reduces rahmonic distinguishability in the cepstrum). Although CPPS is affected by both *f*_o_ and SPL [7,9,39], no effect was seen in our data, likely owing to the controlled nature of the tasks in our protocol (‘soft’ singing on a prescribed frequency).

Vibrato perturbation was measured using the *BioVoice* V_Jitt_ and V_Shim_ parameters. In our study, V_Jitt_ values ranged from 2.43 to 37.3% (*M* = 11.59, *SD* = 8.59). V_Shim_ ranged from 11.4 to 47.95% (*M* = 27.27, *SD* = 11.55). These mean values are slightly higher than those reported in Manfredi et al. [46] but may result from task differences. In their study, singers were asked to perform a standardized melody in a comfortable key and volume from which one sustained tone was analyzed. In the present study singers sustained a quietly sung /a/ vowel on a prescribed *f*_o_. Thus, lower P_Sub_ may have contributed to decreased vocal stability in our participants [106].

Vibrato is a significant feature of the WCSV and a common element in neutral CCM singing, the regularity and freedom of which is a mark of skilled and healthy singing voice production [63,64,107]. Several studies have identified regularity in VR and VE as important characteristics in perceptual rating tasks performed by both naïve and expert listeners. Ekholm et al. [108] found that a delay in vibrato onset was negatively associated with perceived vibrato appropriateness (rated by seven expert voice teachers). Anand et al. [109] found a relationship between *f*_o_, VR and VE, and vibrato appropriateness as rated by four experts and five student judges. While this appropriateness was related to pedagogical and musico-aesthetic ideals, it is also of relevance to the present study. Small changes or instability in VR and VE evidently bear weight in perceptual judgement of vibrato, and these may have greater weight in self-assessment of singing function than smaller perturbations that are reflected in short-term perturbation measures (e.g., APQ3).

Although VE can be adjusted through training [43,44,110,111], no training effect was seen in our data, despite the large range of years of training in our participants. The use of the V_Jitt_ and V_Shim_ parameters somewhat reduces the potential confounding influence of training (where VR and VE can vary greatly across genres). Regardless of the VR or VE, if the vibrato is stable lower V_Jitt_ and V_Shim_ values should reflect such. It would be inappropriate for a clinician or researcher to request a singing participant to regulate their VR or VE for the sake of the voice analysis. Thus, vibrato-perturbation-related measures show great promise for singing voice analysis, allowing for application across genres and for intersubject and pre/posttreatment comparisons, ergo between singers with different vibrato rates and extents.

The relationships between V_Jitt_, V_Shim_, and voice condition, particularly vocal fatigue, is somewhat intuitive. As free vibrato originates in part through quasi-sinusoidal oscillatory contractions of the cricothyroid muscle (i.e., an oscillating *f*_o_), muscle fatigue or vocal fold swelling may interfere with vibrato regularity. As a free vibrato involves a complex interaction between pressures, flows, resonances, and neuromuscular systems [43,112,113], measuring vibrato stability offers more detail about singing-voice function and condition than independent VR or VE values. It is possible that the same factors that contribute to huskiness or breathiness on high notes (e.g., reduced vocal fold adduction and motility through swelling or fatigue of adductory muscles) also affect vibrato stability.

### 4.3. Perceptions of Singing Voice Fatigue and the Efficiency Ratio

As singers are trained to proprioceptively evaluate their vocal function, small changes in their ability to perform specific vocal tasks (e.g., in efficiency) may be relevant contributors to their perception of vocal fatigue. Toles, Seidman, et al. [48] found the ER (SPL/P_Sub_) to be sensitive pre/post excision of phonotraumatic lesions. They reported a mean ER of 9.25 (*SD* = 2.12) measured post-surgery during /papapapapa/ phonation at a comfortable pitch and volume. In the present study, participants’ ER values ranged from 7.78 to 15.90 (*M* = 10.60, *SD* = 2.35). As classical singers are trained to optimize vocal efficiency, the ER maximum of 15.9 reached in our cohort is not surprising.

Titze [91] defined glottal efficiency as the ratio between aerodynamic input and acoustic output (p. 269). As unamplified voice production remains the norm in Western classical singing, finding maximum acoustic output with relatively minimal effort is key to maintaining sustainable and healthy (i.e., non-pressed) phonation. One potential limitation of P_Sub_-based efficiency ratios is that, to a point, a high P_Sub_ and a well-adducted glottis will usually improve ER [63,114]. Thus, it may be difficult to distinguish between efficient (and sustainable) and hyperfunctional phonation solely based on ER. Further, *f*_o_ influences ER, as higher *f*_o_ are stronger in SPL owing to resonance-harmonics interactions and greater radiation efficiency [70,91,115,116]. Previous research has noted an increase in speakers’ PTP after increased vocal demand [90,97,117], most likely owing to increased tissue viscosity, thickness of the vocal folds’ colliding edge, and sub-optimal (i.e., too narrow or too wide) prephonatory glottal width [117,118,119]. Inadequate glottal adduction reduces acoustic power (i.e., ER), whereas high medial compression with P_Sub_ in the realm of pressed phonation would increase ER. A challenge then lies in identifying the line between practical (sustainable) singing-voice efficiency and potential hypertension represented in elevated ER values.

In our study, the use of soft phonation at a standardized *f*_o_, and the inclusion of other voice-source-related acoustic measures may have somewhat mitigated this potential confounding influence: no dB or *f*_o_ effects were found. Soft phonation is used in the voice clinic (and studio) as an indication of not only behavioral adjustment, but also voice condition [120,121]. The ability to maintain adequate prephonatory glottal approximation for ease of oscillatory initiation as well as a relatively fast closing phase (i.e., improved power to output ratios), whilst simultaneously reducing intensity (dB) is a maneuver that requires fine muscle coordination challenging for the fatigued or otherwise dysfunctional singing voice [122,123]. The associations between ER and the scale items related to perceived huskiness (Q1) and breathiness (Q11) highlights this. The significant contribution of ER in the ASFI model offers support for the inclusion of aerodynamic measures (complementary to acoustic measures) in an instrumental index for diagnosing functional changes in singers with singing-voice complaints.

### 4.4. Limitations and Future Directions

To the authors’ knowledge, this exploratory study represents the first attempt to explicitly examine relationships between the individual EASE scale items and instrumental aeroacoustic voice measures. The data presented illustrate how an instrumental index that relates to singers’ nuanced perceptions of their singing-voice function can be constructed.

As the sample size in this study was small, it would be premature to widely generalize the findings. Future studies would benefit from larger cohorts of both normophonic and dysphonic singers. While useful for exploring possible associations in a novel field, we acknowledge that the use of stepwise regression with backward elimination may have excluded some relevant parameters. This statistical approach is widely used in contemporary research (e.g., [124,125,126,127]), and here provides some proof-of-concept supporting further validation research, which should utilize a wider range of analyses including permutation statistics, applied to larger datasets.

Some functional and perceptual changes in the singing voice may be traceable only intrasubject. For instance, given the wide range of norms within the human voice, one singer’s baseline healthy measurements may be approaching dysphonic for another. It would be beneficial to compare intrasubject changes in the ASFI over time and after various levels of vocal demand. No instrumental measures were related to the *PRI* or *VC* subscales, and the ASFI showed only a moderate association with the *PRI* (*p* < 0.10). Further research with populations including disordered singers may clarify these relationships.

The use of the PAS with its mask may have somewhat altered the singing voice function of participants. Future research should explore less intrusive methods of collecting pressure data. More work is needed to determine which existing tools are suitable for use with the singing voice, and to develop robust, standardized singing-voice assessment protocols that can be implemented in clinical and pedagogical contexts. Although the instrumental measures explored in the present study represent a broad range across time, frequency, and aerodynamic domains, they are by no means exhaustive. Future research may benefit from exploring relationships between self-perception of singing voice function and health status and tools such as the voice range profile or non-linear analyses.

## 5. Conclusions

Subtle changes in singing-voice function are sensed by skilled voice users but are not always perceived aurally by a third party or readily identified using existing instrumental voice assessment techniques. Despite being a high-risk population, no standardized clinical protocol for singing voice analysis exists and little research has been carried out to determine the suitability of traditional clinical voice diagnosis approaches for use with the singing voice. Thus, singing-voice complaints presented by the professional voice user that indicate early signs of dysfunction may go undiagnosed until their severity progresses. This leaves a large gap in the care of professional singing-voice users who rely on optimal vocal function for livelihood. This exploratory study offers novel data illustrating associations between EASE scale items and instrumental aeroacoustic measures. Three of the 22 original items were correlated with instrumental voice measures, the composite score of which was significantly associated (𝜏 = 0.742, *p* < 0.0001) with the *VF* subscale. Multiple linear regression techniques indicated that CPPS, V_Jitt_, V_Shim_, and ER (measured during soft sung phonation) accounted for 62.4% of variation in the combined scores of the three scale items. This instrumental index was also significantly associated (𝜏 = 0.575, *p* = 0.001) with the original *VF* subscale. These instrumental measures show promise for singing voice analysis individually and as part of an instrumental index as illustrated here. Further development of diagnostic protocols for singers is needed for preventative and rehabilitative healthcare for professional singing-voice users.

## Figures and Tables

**Table 1:** Descriptive Statistics (*N* = 18) for All Included Instrumental Variables.

Measure	Min	Max	Mean	*SD*
NAQ ([l/s]^2^)	0.131	0.301	0.209	0.042
RAP (%)	0.032	0.448	0.129	0.124
APQ3 (%)	0.204	1.83	0.727	0.418
CPPS (dB)	11.58	18.02	14.40	1.88
V_Jitt_ (%)	2.43	37.30	11.59	8.59
V_Shim_ %	11.40	47.95	27.27	11.55
ER (dB/cm H_2_O)	7.78	15.90	10.74	2.34

Note. NAQ: Normalized Amplitude Quotient. RAP: Relative Average Perturbation. APQ3: Amplitude Perturbation Quotient 3. CPPS: Smoothed Cepstral Peak Prominence. V_Jitt_: Vibrato Jitter. V_Shim_: Vibrato Shimmer. ER: Efficiency Ratio.

**Table 2:** Regression Model for the EASE-3.

	Estimate	Std. Error	T Value	Sig.
Intercept	14.0114	2.93662	4.771	<0.000 ***
CPPS (dB)	−0.41753	0.17085	−2.444	0.029 *
V_Jitt_ (%)	0.10822	0.03039	3.561	0.003 **
V_Shim_ (%)	0.1164	0.02927	3.977	0.002 **
ER (dB/cm H_2_O)	−0.6764	0.14007	−4.829	<0.000 ***
Residual std. error: 1.014 on 13 *df*		Adjusted R^2^ = 0.624		
F-statistic: 8.064 on 4 and 13 *df*		*p* = 0.001696 **		

Note. EASE-3: Evaluation of Ability to Sing Easily-3. CPPS: Smoothed cepstral peak prominence. V_Jitt_: Vibrato jitter. V_Shim_: Vibrato shimmer. ER: Efficiency ratio. Significance: *** *p* < 0.001, ** *p* < 0.01, * *p* < 0.05.

## Data Availability

Participant recordings have not been made publicly available to protect confidentiality. Further inquiries can be directed to the corresponding author.

## Appendix A

Full list of items included in the Evaluation of Ability to Sing Easily, from Phyland et al., 2014 [15]. *VF*: Vocal fatigue. *PRI*: Pathological risk indicators. *VC*: Voice concerns.

1. My voice is husky (*VF*)
2. My voice is dry/scratchy (*VF*)
3. My voice cracks and breaks (*PRI*)
4. My throat muscles are feeling overworked (*VF*)
5. My voice is breathy (*PRI*)
6. My singing voice feels good (*VF*; reverse scored)
7. The onsets of my notes are delayed or breathy (*VF*)
8. My voice feels strained (*VF*)
9. I am worried about my voice (*VC*)
10. I am having difficulty with my breath for long phrases (*PRI*)
11. My top notes are breathy (*VF*)
12. My voice sounds rich and resonant (*VF*)
13. My voice is cutting out on some notes (*PRI*)
14. I am having difficulty singing softly (*PRI*)
15. My voice is tired (*VF*)
16. I am having difficulty changing registers (*PRI*)
17. I am having difficulty with my high notes (*PRI*)
18. Singing feels like hard work (*PRI*)
19. I am having difficulty projecting my voice (*PRI*)
20. I am concerned about my voice (*VC*)
21. My voice feels ready for performance if required (*VF*; reverse scored)
22. I am having difficulty sustaining long notes (*PRI*).
